# Neuroprotective Effects of the Cultivated *Chondrus crispus* in a *C. elegans* Model of Parkinson’s Disease

**DOI:** 10.3390/md13042250

**Published:** 2015-04-14

**Authors:** Jinghua Liu, Arjun H. Banskota, Alan T. Critchley, Jeff Hafting, Balakrishnan Prithiviraj

**Affiliations:** 1 Department of Environmental Sciences, Faculty of Agriculture, Dalhousie University, P.O. Box 550, Truro, NS B2N 5E3, Canada; E-Mail: j.liu@dal.ca; 2 Aquatic and Crop Resource Development, National Research Council Canada, 1411 Oxford Street, Halifax, NS B3H 3Z1, Canada; E-Mail: Arjun.Banskota@nrc-cnrc.gc.ca; 3 Acadian Seaplants Limited, 30 Brown Avenue, Dartmouth, NS B3B 1X8, Canada; E-Mails: Alan.Critchley@acadian.ca (A.T.C.); jhafting@acadian.ca (J.H.)

**Keywords:** *Chondrus crispus*, neuroprotective effect, oxidative stress, Parkinson’s disease

## Abstract

Parkinson’s disease (PD) is the second most common neurodegenerative disorder in the elderly people, currently with no cure. Its mechanisms are not well understood, thus studies targeting cause-directed therapy or prevention are needed. This study uses the transgenic *Caenorhabditis elegans* PD model. We demonstrated that dietary supplementation of the worms with an extract from the cultivated red seaweed *Chondrus crispus* decreased the accumulation of α-synulein and protected the worms from the neuronal toxin-, 6-OHDA, induced dopaminergic neurodegeneration. These effects were associated with a corrected slowness of movement. We also showed that the enhancement of oxidative stress tolerance and an up-regulation of the stress response genes, *sod-3* and *skn-1*, may have served as the molecular mechanism for the *C. crispus*-extract-mediated protection against PD pathology. Altogether, apart from its potential as a functional food, the tested red seaweed, *C. crispus*, might find promising pharmaceutical applications for the development of potential novel anti-neurodegenerative drugs for humans.

## 1. Introduction

Parkinson’s disease (PD) is the second most common neurodegenerative disorder in the elderly people. PD is characterized by selective loss of dopaminergic (DAergic) neurons [1] resulting in the patients having motor and recognition complications, including tremor and slow movement. Although medication and surgery are available for treatment to alleviate the symptoms, there is currently no cure. Through linkage analysis, genome-wide association studies, and candidate gene approaches, α-synuclein (α-syn) and several other genes have been identified to be associated with PD [2]. The *α-syn* gene encodes a small pre-synaptic protein which has the propensity to aggregate and is found in Lewy bodies, the pathological hallmark of PD [3]. Over-expression of α-syn or mutations in this gene result in early-onset of the rare familial forms of PD. More importantly, α-syn is also implicated in the more common sporadic forms [4]. Increased levels of α-syn protein lead to neurodegeneration in both mouse and *C. elegans* models [5,6]. To efficiently treat and/or prevent PD, further investigations are required to discover the mechanism of the disease and cause-directed therapy.

The free-living nematode *Caenorhabditis elegans* has a conserved DAergic system and a simple and well-described nervous system consisting of 302 neurons [7]. These features make *C. elegans* an ideal model for neuroscience research. Over-expression of the human α-syn, which was fused to YFP (yellow fluorescent protein), in *C. elegans*, allows for visual detection of α-syn aggregation by a fluorescent microscope [8]. Moreover, transgenic strains of *C. elegans* expressing GFP (green fluorescent protein) in the DAergic neurons enable the fate of neurons upon DAergic neuron-degenerative drug treatment to be monitored [9,10].

The red seaweed *Chondrus crispus* (Rhodophyta), commonly known as Irish Moss, is widely distributed in the northern Atlantic and harvested as a raw material for the extraction of carrageenan, which finds wide applications in food and cosmetic industries as thickeners, stabilizers and emulsifiers. Besides a relatively high content of the sulfated polysaccharide carrageenan, this red alga is rich in proteins, peptides, amino acids, lipids and pigments; all of which can impart various health benefits to humans, including neuroprotective activity [11,12,13]. In the present study, we used the α-syn:YFP transgenic *C. elegans*, as well as a transgenic strain expressing GFP in the DAergic neurons in order to investigate the neuroprotective effects of an extract from the cultivated red seaweed, *C. crispus*.

## 2. Results

### 2.1. Effects of the Methanolic Extract of Chondrus crispus (CCME) on the General Health of C. elegans

**Figure 1:**
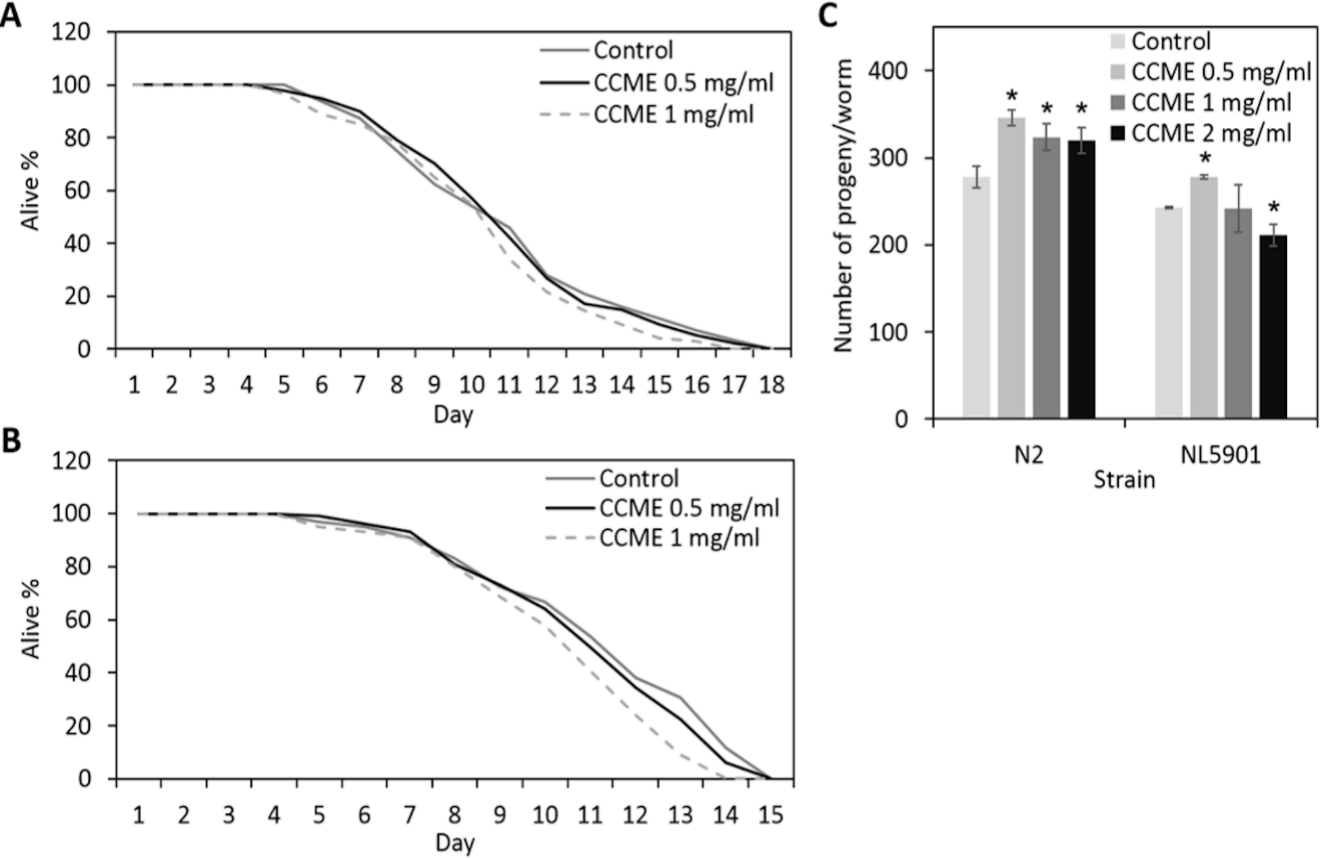
Effect of dietary supplementation of CCME on the lifespan and reproduction of *C. elegans.* (**A**) Effect on the lifespan of the wild type strain N2; (**B**) Effect on the lifespan of the α-syn transgenic strain NL5901; (**C**) Effect on the brood size of N2 and NL5091 strains. Worms were supplemented with 0 (control), 0.5, 1, or 2 mg/mL of CCME from L1 stage throughout their life, and the survival percentage of worms was scored daily until all worms were dead (*n* = 100–150/treatment). Data were presented as the mean ± standard deviation from three independent experiments. * Values are significantly different from the control (*p* < 0.05). CCME, *Chondrus crispus* methanolic extract.

In an attempt to test whether CCME affected the general health of the animals, we carried out lifespan and brood size assays with 0, 0.5, 1.0, or 2.0 mg/mL of CCME supplemented to the standard laboratory nematode growth medium (NGM). As shown in Figure 1, in both the wild type strain N2 (Figure 1A) and the transgenic strain NL5901 (Figure 1B), the lifespan of treated worms was not significantly affected, as compared to the untreated control. We also observed that the pace of the development of the worms was identical across treatments (data not shown). Furthermore, CCME increased the brood size, especially at the lower concentration of 0.5 mg/mL (Figure 1C). On the other hand, the higher concentration of 2.0 mg/mL either showed less increase of the progeny number for N2 worms, or a noticeable decrease in progeny of the transgenic strain NL5901. Thus, CCME, at the concentrations of 0.5 or 1.0 mg/mL, did not adversely affect the general health of the animals, with the worms showing an unchanged pace of development and lifespan and with significantly larger brood size. Therefore, we used the lower concentration of 0.5 mg/mL for further assays.

### 2.2. CCME Decreased α-syn Accumulation

**Figure 2:**
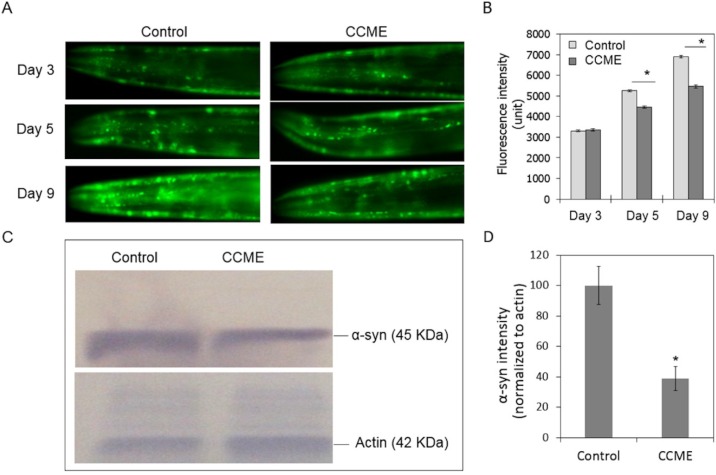
Dietary supplementation of CCME to worms decreased α-syn accumulation. (**A**) Representative images of α-syn accumulation. Synchronized L1 worms of the transgenic strain NL5901 was dietary supplemented with CCME (0, 0.5 mg/mL), cultured until 3, 5, or 9 days of adulthood. Fluorescent images were taken for the head region each worm (*n* = 90–100/treatment); (**B**) Quantification of α-syn from YFP fluorescence. The YFP intensity in the head region, which represents the accumulated α-syn protein, was analyzed with the ImageJ software and compared between the control and the CCME-treated groups. (**C**) Western blots of α-syn protein. On day 9 of adulthood, NL5901 worms, cultured in the presence or absence of 0.5 mg/mL CCME in their diet from the L1 stage, were subjected to Western blot analysis using the YFP antibody to detect the conjugated α-syn protein. Blots of actin were used as a protein loading control; (**D**) Quantification of α-syn from Western blots. The intensity of protein bands was quantified using the ImageJ software. The intensity of α-syn was normalized to actin. Data represented two independent experiments. For each treatment of each experiment, protein samples were pooled from three biological replicates. Data were presented as the mean ± SD. * *p* < 0.05. CCME, *Chondrus crispus* methanolic extract.

We then utilized the transgenic NL5901 strain of *C. elegans*, which constitutively expresses YFP-fused human α-syn protein in the body wall, to study the effect of CCME. CCME was supplemented to the normal diet of synchronized L1 worms until the 9th day of adulthood. At 3, 5, or 9 days of adulthood, worms (*n* = 50–70/treatment) were observed under a fluorescence microscope to visualize the α-syn deposition in the head region. As shown in Figure 2A,B, on day 3, the accumulated α-syn was comparable between the control and the CCME-treated group. On day 5, a pronounced decrease of α-syn was observed in the CCME group (*p* < 0.05), as compared to the control. Remarkably, on day 9, α-syn accumulation in the control worms was significantly increased, whereas this increase was less in the CCME-treated group, resulting in a notable reduced intensity of fluorescence in the CCME group, as compared to the control (*p* < 0.05). To further confirm that CCME decreased α-syn accumulation, we performed Western blot analysis, using antibodies detecting the YFP part of the α-syn::YFP chimeric protein, to quantify the α-syn protein in whole worms (not only the head region), on the 9th day of adulthood. Consistent with the fluorescence microscopy data, we observed a marked decrease (*i.e.*, 61%) of the α-syn protein level in the CCME treated worms (*p* < 0.05), as compared to the control (Figure 2C,D). Thus, the accumulation of α-syn protein, a critical process in PD development in humans, was decreased with dietary supplementation with CCME, in the *C. elegans* tested.

### 2.3. CCME Protected C. elegans from Drug-Induced DAergic Neuron Degeneration

**Figure 3:**
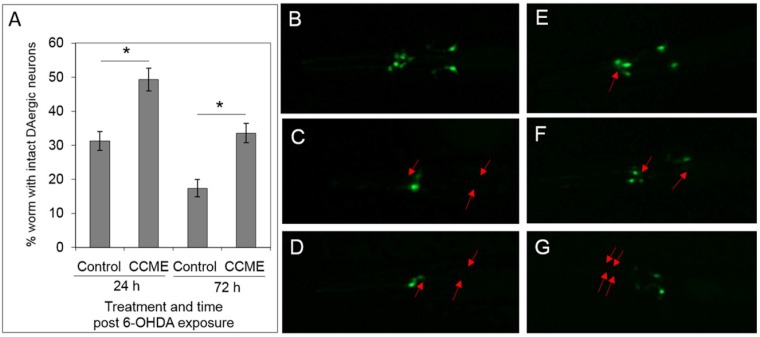
CCME protected *C. elegans* from 6-OHDA induced DAergic neuron loss. (**A**) Quantification of worms with intact DAergic neurons. The L1 worms of the transgenic strain UA57, with GFP expression in the DAergic neurons, which had been raised in the presence or absence of CCME from synchronizied eggs, were exposed to the neurotoxin 6-OHDA prior to further cultured for 24 or 72 h in the presence or absence of CCME. Images of the head region of each worm were then taken under a fluorescence microscope, with the GFP signals showing the DAergic neurons (*n* = 100–150/treatment). Representative images were shown for the head region of worms with intact DAergic neurons (**B**), and various patterns of loss of one or more DAergic neurons (**C**–**G**), with the red arrows showing the missing/degenerated neurons. Data were presented as the mean ± SD. * *p* < 0.05. CCME, *Chondrus crispus* methanolic extract.

As with mammals, 6-OHDA was previously shown effectively degenerate the DA neurons in *C. elegans*, through oxidative stress [9]. Here we tested the protective effect of CCME in this drug-induced neuron degeneration using a *C. elegans* strain (UA57) expressing GFP in DAergic neurons. At 24 h post-exposure of the synchronous L1 (lava stage 1) worms to 6-OHDA, CCME-treated worms showed the highest percentage of intact DAergic neurons, as compared to the controls (Figure 3). The trend remained the same at 72 h post 6-OHDA exposure, with 34% of the worms with intact DAergic neurons in the CCME-treated group, but only 18% of the worms with intact DA neurons in the control group (*p* < 0.05).

### 2.4. CCME Supplementation Prevented the Rapid Slow-down of Body Movement upon Treatment with 6-OHDA

**Figure 4:**
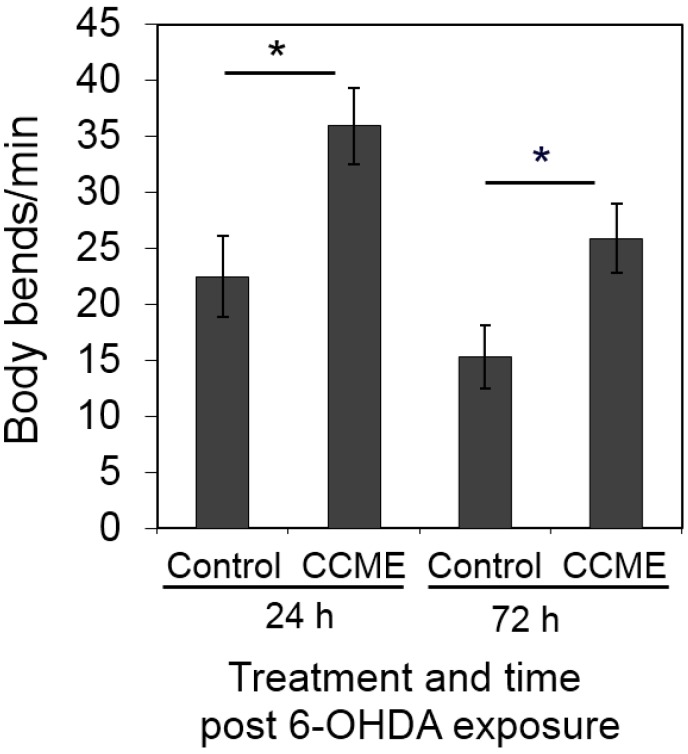
Effect of CCME on the locomotion of transgenic worms. The L1 worms of the transgenic strain UA57, with GFP expression in the DAergic neurons, which had been raised in the presence or absence of 0.5 mg/mL of CCME from synchronized eggs, were exposed to the neurotoxin 6-OHDA prior to further cultured for 24 or 72 h in the presence or absence of 0.5 mg/mL of CCME. The locomotion of each worm were examined by counting the number of body bends per min (*n* = 30–50/treatment). Data were presented as the mean ± SD. * *p* < 0.05. CCME, *Chondrus crispus* methanolic extract.

The 6-OHDA-induced DAergic neuron degeneration in transgenic *C. elegans* is associated with a significant reduce in locomotion overtime [9]. Thus, we also studied the effect of CCME on the locomotion of AU 57 transgenic worms, post-6-OHDA treatment. At 24 h post exposure of the synchronous L1 worms to 6-OHDA, CCME-treated worms showed the faster body movement of 35 body bends per min, as compared to the control (23 bends per min) (Figure 4). At 72 h post-6-OHDA exposure, the CCME-treated worms crawled at a speed of 27 body bends per min, while the control worms moved significantly more slowly at 15 body bends per min (*p* < 0.05, *n* = 30–50/treatment) (Figure 4). Therefore, in the 6-OHDA-induced DAergic neuron degeneration system, dietary supplementation of CCME was observed to prevent the dramatic slowdown in locomotion observed at both 24 h and 72 h post-6-OHDA exposure.

### 2.5. The Decreased α-syn Accumulation by CCME Supplementation Was Associated with Enhanced Tolerance of Oxidative Stress, but not Heat Stress

**Figure 5:**
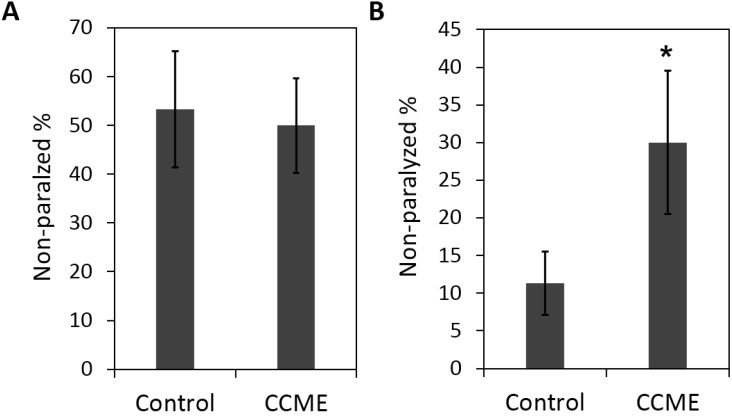
Effect of CCME on the tolerance of heat stress (**A**) and oxidative stress (**B**). The transgenic worms (strain NL5901) expressing human α-syn were raised from synchronized L1 stage, in the presence or absence of CCME as a diet supplement. On day 5 of adulthood, worms were exposed to either heat stress of 30 °C or a juglone-induced moderate oxidative stress. Percentage of worms that was not paralyzed were evaluated. The experiments were repeated three times. Data were presented as the mean ± SD. * *p* < 0.05 (CCME treated *vs.* control). CCME, *Chondrus crispus* methanolic extract.

Multiple factors, including oxidative stress and other environmental factors may play roles in the etiology of PD. Here we investigated the effect CCME on the tolerance of heat and oxidative stresses in α-syn transgenic worms (strain NL5901). Worms were raised from the synchronized L1 stage, in the presence or absence of CCME as a dietary supplement. On the 5th day of adulthood, worms were exposed to either heat stress or a juglone-induced, moderate oxidative stress. As shown in Figure 5A, dietary supplementation of CCME at 0.5 mg/mL had no effect on the heat stress tolerance of the worms, with the non-paralyzed numbers being 54% for the control and 49% for the 0.5 mg/mL CCME group (*p* > 0.05, treatment *vs.* control). However, the 0.5 mg/mL CCME was shown to enhance the oxidative stress tolerance with 32% non-paralyzed worms, while the control had 12% worms which were not paralyzed (*p* < 0.05) (Figure 5B).

### 2.6. The Decreased α-syn Accumulation by CCME Was Associated with the Up-Regulation of the sod-3 and skn-1Genes

**Figure 6:**
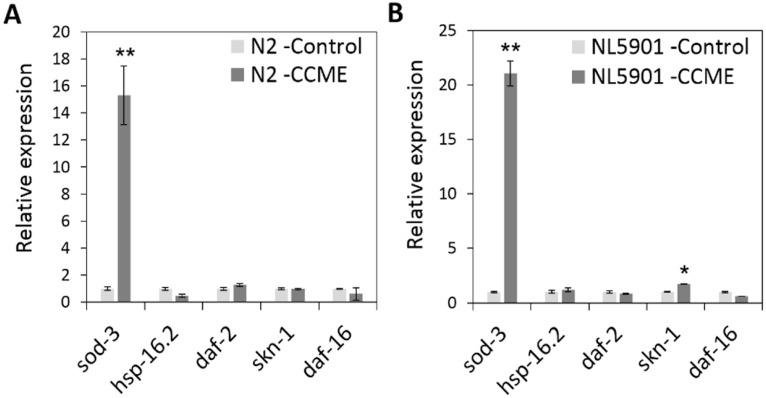
Effect of CCME on the expression of stress response genes in the wild type N2 (**A**) and the transgenic NL5901 strains (**B**). The worms were cultured in the presence of absence of CCME as a dietary supplement from L1 stage. On day 5 of adulthood, gene expression level was analyzed using qPCR. The data represents three independent experiments. Data were presented as the mean ± SD. ** *p* < 0.01; * *p* < 0.05 (CCME *vs.* control). CCME, *Chondrus crispus* methanolic extract. qPCR, quantitative polymerase chain reaction.

To explore the molecular mechanism for the CCME-elucidated, protective effect, we performed gene expression analysis of several oxidative stress /heat stress/immune response genes, including *sod-3*, *hsp-16.2*, *daf-2*, *daf-16* and *skn-1*. The worms were cultured with 0 or 0.5 mg/mL of CCME as a dietary supplement from L1 stage. On day 5 of adulthood, the expression level of the genes was analyzed using qPCR. For the wild type N2 worms, the oxidative response gene, *sod-3*, showed a 15-fold up-regulation in the CCME-treated group, as compared to the control (*p* < 0.01) (Figure 6A). In α-syn transgenic worms (strain NL5901), a 22-fold up-regulation of the oxidative response gene, *sod-3*, was evident in the CCME-treated worms, as compared to the control (*p* < 0.01) (Figure 6B). Moreover, a 1.8 fold up-regulation of *skn-1* was observed with CCME supplementation, as compared to the control (*p* < 0.05). However, CCME did not affect the expression of other genes analyzed, such as *hsp-16.2*, *daf-2* and *daf-16*.

## 3. Discussion

In the present study, we determined that a methanolic extract from the cultivated red seaweed *Chondrus crispus* (CCME) decreased the accumulation of the human PD protein α-syn in transgenic *C. elegans*. Since accumulation of α-syn is toxic, resulting in neuronal degeneration, in various PD models [5,6], our observations of the decreased accumulation of α-syn, in the present study, suggested a promising neuroprotective effect of CCME. This result was further validated by our observations that CCME protected the DAergic neurons from rapid degeneration and prevented slowness of movement in a drug-(6-OHDA)-induced PD model of GFP-transgenic worms. Besides *C. crispus*, several seaweeds have been shown to have neuroprotective effects. For instance, fucoidan extracted from the brown alga *Fucus vesiculosus*, was reported to protect rat cholinergic neurons from the beta amyloid peptide-, Aβ1-42-induced cell death [14]. Additionally, two seaweeds, *Hypnea valentiae* (red) and *Ulva reticulata* (green) were reported to have neuroprotective activity in Alzheimer’s disease models [15]. Yoon *et al.* [16] screened 27 marine algae, and found that extracts from the brown kelp *Ecklonia stolonifera* showed inhibitory activity to neuronal cell death. Apart from seaweeds, natural products derived from terrestrial plants have also been reported with neuroprotective effects, although the mechanisms largely remain unknown. For example, the Ginkobiloba leaf extract (EGb761), which contains flavonoids, organic acids and terpenoids, has been used as a supplement to improve memory and reduce age-related neuronal deterioration [17,18]. In addition, dietary supplementation with blueberry was demonstrated to enhance memory and motor performance in aged animals [19,20]. These health benefit of blueberry was thought to be attributed to the accumulation of the predominant antioxidant in blueberry, anthocyanin, in the hippocampus and neocortex region of the brain [21]. Moreover, the common spices, garlic (*Allium sativum*) and hot pepper (*Piper longum*) were found to either improve the hippocampal-based memory deficit, or the cognitive performance in both AD patients [22] and rats [23].

The CCME sample used in the present study comprises of floridoside, isethionic acid, taurine, unsaturated fatty acids, phenylalanine and l-cirtuline [13]. Number of galactolipids, lutein, eicosapentaenoic acid (EPA) and arachidonic acid (AA) were also isolated and identified from the methanolic extract of *C. crispus* possessing strong anti-inflammatory properties [24]. Floridoside and isethionic acid are the major components of CCME comprising 7.51% and 9.08%, respectively (Sangha *et al.* unpublished) [25] and both have antioxidant potential [26]. Recently, Kim *et al.* [27] reported that floridoside has the ability to suppress pro-inflammatory responses in lipo-polysaccharide (LPS)-activated microglia cells via blocking p38 and MAPK signaling pathways, suggesting that floridoside may be a potential agent against neuroinflammation-mediated, neurodegeneration. Similarly, taurine, an abundant amino acid in the retina, showed neuroprotective effects on retinal ganglion cells [28] and have protective effects against glutamine-induced, neuronal injury in cultured neurons [29]. Isethionic acid, an analogue of taurine without the amino group, was also reported to potentiate dihydrorhodamine 123 (DHR) oxidation by 3-morpholinosydnonime [30]. Lutein and EPA were also reported to have neuroprotective effects in retina- [31] and LPS- induced dysfunction in rat hippocampus [32], respectively. In addition, unsaturated fatty acids and glycolipids were found to alleviate oxidative stress and thus have antioxidant activity [13,33,34]. Nevertheless, due to the fact that CCME consists of multiple bioactive compounds and each of them constituents a small proportion, the CCME-imparted neuroprotective effect in *C. elegans* is most likely to be a synergistic effect of the unsaturated fatty acids, glycolipids, floridoside, isothionic acid and other components such as pigments and free amino acids. Interestingly, the major component of *C. crispus*, κ-carrageenan, although not present in CCME, was recently shown to have potentials for anti-inflammation [35] and preventing the neurodegenerative processes [36].

In the present study, we found that dietary supplementation of CCME enhanced the oxidative stress tolerance in *C. elegans*, and this was associated with the up-regulation of the stress response genes *sod-3* and *skn-1*. Coincidently, CCME was reported to mitigate oxidative stress in non-PD related strains of *C. elegans* [13]. Oxidative stress is thought to be an underlying mechanism for the pathology of PD of both sporadic and familial forms [37]. In line with this, increases in the oxidized lipids [38] and proteins and DNA [39] were observed in the brain of PD patients. DAergic neurons are particularly sensitive to oxidative stress, which is associated with DA metabolism, mitochondria dysfunction, and neuroinflammation, thus, a moderate oxidative stress can trigger a cascade of cellular reactions and cause neuronal cell death [37]. As a result, functioning against oxidative stress, agents with antioxidant activity, such as CCME, are suggested to be protective to DAergic neurons, and thus alleviate PD pathology. Here, we identified a 22 fold up-regulation of the antioxidant effector gene, *sod-3*, with CCME supplementation, suggesting the role of *sod-3* in the neuroprotective effect, through an anti-oxidative pathway. *C. elegans* has five superoxide dismutase (sod) genes; *sod-1* and *sod-5* are expressed in the cytoplasm; *sod-2* and *sod-3* are expressed in the mitochondria, while *sod-4* is extracellular. Recently, one of the two mitochondrial sod genes, *sod-3*, was shown to be up-regulated in the presence of antioxidants, and inhibited oxidative stress-induced DNA damage [40]. In another study, chemical components of *C. crispus* upregulated *sod-3* [13]. Therefore we tested the effect of CCME on *sod-3* expression and hypothesize that an increased expression of *sod-3* contribute to the neuroprotective effect of CCME. Nevertheless, direct verification of the potentially up-regulated *sod-3* protein in DAergic neurons, for example through immunocytochemistry and/or *in situ* hybridization assays, are suggested for future studies. Moreover, we observed a significant increase in the gene expression of *skn-1*, with CCME supplementation, in transgenic *C. elegans* expressing human α-syn; while the expression level of other tested stress-response genes, such as *daf-16*, *daf-2* and *hsp-16.2*, were un-affected. As a transcription factor, *skn-1* positively regulates the transcription of free radical-scavenging enzymes and plays an important role in stress resistance [41,42]. Thus, up-regulation of *skn-1*, but not *daf-16*, *daf-2*, or *hsp-16.2*, may serve as another molecular pathway that contributed to the CCME-imparted oxidative stress tolerance and neuro-protection, In line with this, it was recently reported that *skn-1* is critical in protecting *C. elegans* from methylmercury-induced oxidative stress and DAergic neuron loss [43]. Therefore, in our present study, the enhancement of oxidative stress tolerance through up-regulation of the stress response genes, *sod-3* and *skn-1*, may serve as the molecular mechanism(s) for the CCME-induced protection against PD pathology.

## 4. Experimental Section

### 4.1. Seaweed Extract and Chemicals

A methanolic extract of *Chondrus crispus* (CCME) was prepared, as previously described [13] and stored at −20 °C. Briefly, cultivated *C. crispus* was collected, rinsed with distilled water and immediately lyophilized and vacuum sealed. Ten grams of the lyophilized seaweed was extracted with methanol (50 mL × 3), stirring at room temperature for 1 h, and sonicated for 15 min. Excessive solvent was evaporated under reduced pressure, yielding 0.89 g of CCME. For bioassays, a stock solution of 250 mg/mL in methanol was prepared every 2 weeks, stored at 4 °C and diluted to the appropriate concentration in sterile water before use. Dimethylsulphoxide (DMSO) and 6-Hydroxydopamine (6-OHDA) were purchased from Sigma (Oakville, ON, Canada). A working solution of 10 mM 6-OHDA was made with 1% DMSO (v/v) before use.

### 4.2. C. elegans Strains and Maintenance

*C. elegans* strains NL5901 (pkIs2386 [unc-54p::alpha-synuclein::YFP + unc-119(+)]. YFP expression in the muscles), UA57 (baIs4 [dat-1p::GFP + dat-1p::CAT-2]. GFP expression in CEP, ADE and PDE neurons) and wild type N2, as well as their food source, *Escherichia coli* strain OP50, were provided by the Caenorhabditis Genomic Center (CGC), which is funded by NIH Office of Research Infrastructure Programs (P40 OD10440). OP50 *E. coli* were cultured overnight at 37 °C in Luria-Bertani (LB) broth, concentrated by centrifugation at 3500× *g* for 10 min, and stored at 4 °C until use. The *C. elegans* strains were maintained at 20 °C on 1.2% solid nematode growth medium (NGM), which was pre-seeded with 50 μL of live OP50 *E. coli* as a food source, following standard procedures [44].

### 4.3. Phenotype Assays of C. elegans

#### 4.3.1. Lifespan Assay

The experimental plates were prepared by adding the CCME stock solution to various concentrations (*i.e.*, 0, 0.5, 1, 2 mg/mL) to NGM just before use. The NGM plates were then seeded with live *E. coli* OP50 to establish a bacterial lawn as food for the nematode. The synchronized larva stage 1 (L1) worms (*n* = 100–150/treatment) were transferred onto treatment plates and maintained at 20 °C. During the reproduction period, young adult worms were transferred to fresh treatment plates every 2 days to screen out progeny. Thereafter, nematodes were transferred to new treatment plates every 3 days, until all worms were dead. Survival was evaluated daily and the animals were scored as dead if they fail to respond to gentle, repeated touches with a platinum pick. The first day of adulthood was considered as day 1 of age. Individuals that crawled off the walls of the plates and died from desiccation were excluded from the analysis.

#### 4.3.2. Brood Size Assay

Synchronous eggs were obtained by allowing the adults to lay eggs for 2 h on treatment plates, with 0, 0.5, 1 or 2 mg/mL of CCME in the medium, and then incubated at 20 °C for 2 days. The larvae at L4 stage (*n* = 10–15/treatment) were transferred individually to treatment plates for egg laying. The worms were transferred to fresh treatment plates daily, just prior to the number of eggs for each worm was counted under a dissection microscope, until reproduction is completed.

#### 4.3.3. Heat Stress Assay

The transgenic strain NL5901 worms were raised on NGM plates, in the absence (as the control) or presence of 0.5 mg/mL of CCME, from synchronized L1 larvae. The worms were transferred to fresh assay plates very two days. The day 5 adult worms were picked into wells of a 96-well plate with M9 buffer, exposed to 30 °C for 4 h (*n* = 100–150/treatment), then evaluated for paralysis, with the aid of a platinum pick. This experiment was repeated three times. 

#### 4.3.4. Oxidative Stress Assay

The transgenic strain NL5901 worms were raised on NGM plates, in the absence (as the control) or presence of 0.5 mg/mL of CCME, from synchronized L1 larvae. The worms were transferred to fresh assay plates very two days. The day 5 adult worms were picked into wells of a 96-well plate with M9 buffer, containing juglone at concentrations of 0, 25, 50, 100, or 200 µM (*n* = 100–150/treatment). Worms were evaluated for paralysis at 1 h-post exposure to juglone. This experiment was repeated three times.

#### 4.3.5. α-syn Accumulation Assay

CCME (0, 0.5 mg/mL) was supplemented to the diet of synchronized L1 worms of the transgenic strain NL5901. The worms were cultured under standard conditions on NGM plates. At 3, 5 and 9 days of adulthood, the worms were observed under a fluorescence microscope (BioTek, Winooski, VT, USA) at 200× magnification for the YFP signal. Fluorescent images were taken for the head region of each worm (*n* = 90–100/treatment), with the same settings of a digital camera. The YFP intensity in the head region, which represented the accumulated α-syn protein, was analyzed with the ImageJ software and compared between the control (worms without dietary supplementation of CCME) and the CCME-treated group.

#### 4.3.6. The 6-Hydroxydopamine-Induced DAergic Neuron Degeneration Assay

To test the neuroprotective effects of CCME against 6-Hydroxydopamine (6-OHDA)-induced of DAergic neuronal cell death, the protocol published by Nass *et al.* [9] and modified by Marvanova and Nichols [10] was followed, in an attempt to decrease the death of the worms after 6-OHDA exposure, and to elevate the efficiency of the drug for neuronal degeneration. Essentially, synchronized eggs were cultured in the presence or absence of 0.5 mg/mL of CCME till the L1 stage. The L1 worms of strain UA57, with GFP expression in the DAergic neurons, were then washed 3 times with distilled water, transferred to at least 20 volumes of 10 mM 6-OHDA in 1% DMSO, and incubated at RT in the dark for 30 min, with gentle agitation every 10 min. The worms were then washed 3 times with distilled water, transferred to fresh NGM treatment plates with 0 or 0.5 mg/mL of CCME, and cultured at 20 °C for 24 or 72 h before the evaluation of neuronal death. Worms were washed 3 times with M9 buffer, resuspended in M9 buffer, dispensedinto 96-well plates, and observed under a Cytation 3 imaging reader (BioTeK, Winooski, VT, USA), which was equipped with a fluorescence microscope. Fluorescent images were taken for the head region of each worm (*n* = 90–100 /treatment) using the Gen5 2.05 software, and the loss of the GFP signal from the DAergic neurons was used to determine neuronal cell death.

#### 4.3.7. RNA Extraction and Quantification

Total RNA was extracted with the TRIzol reagent (Invitrogen Life) and an RNeasy RNA kit (Qiagen, Toronto, ON, Canada). Briefly, about 100–200 worms were transferred into 100 μL of TRIzol Reagent, homogenized, mixed with 70 μL chloroform and centrifuged at 10,000× *g* at 4 °C for 15 min. The supernatant was mixed with 70 μL ethanol (70%) and loaded onto RNeasy spin columns (Qiagen, Toronto, ON, Canada) to precipitate RNA according to the manufacturer’s protocol. The integrity and quantity of the RNA were assessed by agarose gel electrophoresis and with a NanoDrop ND-2000 spectrophotometer (NanoDrop Technologies, Wilmington, DE, USA). The RNA samples were stored at −80 °C until use.

#### 4.3.8. Real-Time Quantitative PCR (qPCR)

For quantitative gene expression analysis, total RNA samples, derived from three biological replicates of each treatment were pooled. Using a High Capacity cDNA reverse transcription kit (Applied Biosystems, Burlington, ON, Canada), cDNA was synthesized from 2 μg of RNA. Real-time quantitative PCR (qPCR) was performed on a StepOne real-time PCR system (Applied Biosystems, Burlington, ON, Canada) using Promega GoTaq SYBR green reagent (Roche Diagnostics, Mississauga, ON, Canada) with 0.2 μM each gene-specific primer and 10 ng of cDNA as the template. Each pooled sample was run in a reaction mixture with a final volume of 10 μL in triplicate, following the manufacturer’s instructions. The *ama-1* gene was used as an internal control. The primer sequences (5′-3′) are as follows: *sod-3* (AGC ATC ATG CCA CCT ACG TGA; CAC CAC CAT TGA ATT TCA GCG), *hsp16.2* (ACG CCA ATT TGC TCC AGT CT; GAT GGC AAA CTT TTG ATC ATT GTT A), *daf-2* ( GTG GCG TGA GAA TGA AGT GAG; GGA ATT TCG TAG AAT CCG TTG), *skn-1* (AGT GTC GGC GTT CCA GAT TTC; GTC GAC GAA TCT TGC GAA TCA), *daf-16* (TTT CCG TCC CCG AAC TCA A; ATT CGC CAA CCC ATG ATG G), *ama-1* (CTG ACC CAA AGA ACA CGG TGA; TCC AAT TCG ATC CGA AGA AGC).

#### 4.3.9. Protein Extraction and Quantification

Worms were washed 3 times with distilled water, stacked to approximately 20 µL, then homogenized with a disposable pestle for 30 s in 200–300 µL of a lysis buffer (50 mM Tris-HCl pH7.5, 150 mM NaCl, 1 mM EDTA, 0.2 mM DTT, 1% Triton X-100 ,v/v; 10% glycerol, v/v) supplemented with a proteinase inhibitor (1 mM PMSF). The lysate was sonicated for 2 min and centrifuged at 11,000× *g* at 4 °C for 10 min. The supernatant was collected and protein concentration was determined with the BCA assay kit (Pierce Biotechnology, Rockford, IL, USA). The protein samples were stored at −80 °C.

#### 4.3.10. Western Blots

Typically, 20 μg of total protein was mixed with 5× sample buffer (10% SDS, w/v; 20% glycerol, v/v; 0.5% bromophenol blue, w/v; 0.2 M Tris-HCl pH 6.8; 10 mM DTT), heated at 95 °C for 10 min, then loaded in SDS-PAGE gels consisting of a top 4% stacking gel and a bottom 10% resolving gel, and resolved by electrophoresis at 120 V in a running buffer (25 mM Tris, 190 mM glycine, and 0.1% SDS (w/v)). Proteins were electrotransfered to a PVDF membrane (Immobilion-P, Millipore Corporation, Etobicoke, ON, Canada) using a Trans-Blot^®^ SD Semi-dry transfer cell (Bio-Rad, Mississauga, ON, Canada) following the manufacturer’s instructions, and was blocked over night at 4 °C with 5% (w/v) fat-free milk in 1× Tris buffered saline with Tween 20 (TBS-T; 0.05 M Tris-HCl, 0.15 M NaCl, 0.1% (v/v) Tween 20, pH 7.5). The same solution was utilized for the hybridization with the primary antibody (1:1000 dilution, rabbit anti-GFP, Invitrogen Life Technologies, Burlington, ON, Canada) for 3 h. The membrane was then washed 3 times with 1× TBS-T and subsequently probed with a secondary antibody solution (alkaline phosphatase conjugated anti-rabbit; Invitrogen, Burlington, ON, Canada) for 1 h. After three washes with 1× TBS-T, the membrane was developed with the SIGMA*FAST*™ BCIPNBT tablet (Sigma, Oakville, ON, Canada). For re-probing with Actin as the loading control, the membrane was incubated in a stripping buffer (0.1% SDS, w/v; 1% Tween 20, v/v; 200 mM glycine, pH 2.2) for 2 × 10 min, washed twice in 1× TBS then twice in 1× TBS-T, prior to being blocked, hybridized to the primary antibody (1:5000 dilution, rabbit anti-Actin, Sigma, Oakville, ON, Canada) and the secondary antibody and developed, following the manufacturers’ protocols. For each treatment, protein samples from three biological replicates were pooled. The experiment was repeated twice.

#### 4.3.11. Statistical Analyses

Statistical analyses were performed using SPSS software, version 15.0 (Armonk, NY, USA). The data were analyzed using independent student *t* tests when comparing the differences between the treated and the control groups, except for the comparison of survival curve data, which was carried out using the log-rank test of the Kaplan-Meier survival function. Data were presented as the mean ± SD, where applicable. Differences were considered significant when *p* was <0.05.

## 5. Conclusions

Taken together, the results from the this study suggested that a methanolic extract from the cultivated, red seaweed *Chondrus crispus* (CCME) possessed neuroprotective activity, as evidenced by decreased accumulation of α-syn and a lowered DAergic neuron loss in transgenic *C. elegans* models. This protective activity may be attributed to alleviation of oxidative stress and up-regulation of stress response genes, such as *sod-3* and *skn-1*. The multiple compounds in the methanolic CCME extract may function synergistically towards the neuro-protection. Thus, apart from its application as a functional food, the tested red seaweed, *C. crispus*, might find promising pharmaceutical applications for potential novel anti-neurodegenerative drugs for humans.
